# Supplementary material for: Occult Pneumothorax in Blunt Thoracic Trauma: Clinical Characteristics and Results of Delayed Tube Thoracostomy in a Level 1 Trauma Center
Source: J Clin Med. 2023 Jun 28;12(13):4333. doi: 10.3390/jcm12134333 (PMC10342870; doi:10.3390/jcm12134333)
Supplement: Supplementary file 1 [file jcm-12-04333-s001.zip › jcm-2458956-supplementary.pdf]

Supplementary Materials File S1

<Trauma team activation criteria>

1. Physiologic criteria

   A. Airway obstruction/respiratory failure

   B. Intubated status before trauma center arrival

   C. Adults: respiratory rate<10 breaths/min or>30 breaths/min

   D. Adults: systolic blood pressure<90 mmHg

   E. Adults: heart rate>100 beats/min

   F. Glasgow coma scale<13

2. Anatomical criteria

   A. All penetrating injuries

      I. Head and neck, chest, abdomen

      II. Extremity: proximal to elbow or knee

   B. Chest

      I. Flail chest

   C. Nervous system

      I. Open or depressed skull fracture

      II. Paralysis or suspected spinal cord injury

   D. Extremity and pelvis

      I. Pelvic bone fracture

      II. Two or more proximal long-bone fractures

      III. Crushed, degloved, mangled, or pulseless extremity

      IV. Amputation proximal to the wrist or ankle

3. Mechanism of injury

   A. Automobile crash: death in same passenger compartment

   B. Automobile crash: ejection from automobile

   C. Automobile crash>60km/h

D. Automobile versus pedestrian injury>30 km/h

E. Time for evacuation of victim in automobile>20min

(Intrusion, including roof: > 30 cm any site)

F. Motorcycle, bicycle crash > 30km/h

G. Fall

I. Adults: >6m

II. Children: >3m

H. Injury from explosion

4. Attending trauma physician's judgment

* The trauma team should be activated when the patient has more than one criterion.

* The trauma team activation is determined by the findings observed at the beginning of the patient's visit.

Table S1. General characteristics of patients with thoracic trauma according to pneumothorax.

	Total (n = 187)	No pneumothorax (n = 74)	Occult pneumothorax (n = 81)	Overt pneumothorax (n = 32)	*P*-value
Age	55.25 ± 17.60	55.01 ± 20.40	54.23 ± 16.27	58.34 ± 13.54	0.532
Male	143 (76.5)	51 (68.9)	65 (80.2)	27 (84.4)	0.130
Injury mechanism					N/A
Falls	71 (38.0)	34 (45.9)	29 (35.8)	8 (25.0)	
Car accident	50 (26.7)	16 (21.6)	23 (28.4)	11 (34.4)	
Pedestrian accident	36 (19.3)	16 (21.6)	15 (18.5)	5 (15.6)	
Motor/Bicycle	18 (9.6)	4 (5.4)	11 (13.6)	3 (9.4)	
Collision	8 (4.3)	2 (2.7)	3 (3.7)	3 (9.4)	
Assault	1 (0.5)	1 (1.4)	0 (0.0)	0 (0.0)	
Others	3 (1.6)	1 (1.4)	0 (0.0)	2 (6.3)	
RTS	7.54 ± 0.91	7.56 ± 0.96	7.66 ± 0.68	7.16 ± 1.19	0.001*
ISS	19.60 ± 8.72	20.27 ± 8.36	18.37 ± 7.80	21.19 ± 11.28	0.492
ER tube thoracostomy	71 (38.0)	0 (0.0)	39 (48.1)	32 (100.0)	N/A
Tube thoracostomy time (min)	109.1 ± 596.8	0	243.7 ± 892.0	20.5 ± 7.7	0.025*
Positive pressure ventilation	69 (36.9)	23 (31.1)	32 (39.5)	14 (43.8)	0.378
Length of stays (day)	35.35 ± 48.97	31.81 ± 47.82	31.68 ± 39.02	52.81 ± 68.51	0.061
Mortality	3 (1.6)	2 (2.7)	1 (1.2)	0 (0.0)	0.562

Values are presented as mean ± standard deviation or number (%).

*p <0.05; ER-Emergency Room; ISS-Injury Severity Score; min-minute; RTS-Revised Trauma Score.

Table S2. Injury type of chest X-ray according to pneumothorax.

	Total (n = 187)	No pneumothorax (n = 74)	Occult pneumothorax (n = 81)	Overt pneumothorax (n = 32)	*P*-value
Lung contusion	37 (19.8)	11 (14.9)	15 (18.5)	11 (34.4)	0.066
Hemothorax	35 (18.7)	13 (17.6)	17 (21.0)	5 (15.6)	0.799
Subcutaneous emphysema	29 (13.9)	3 (6.8)	13 (16.0)	13 (40.6)	0.000*
Rib fracture	49 (26.2)	11 (14.9)	30 (37.0)	8 (25.0)	0.007*
No. of fractured ribs	0.75 ± 1.43	0.42 ± 1.09	1.05 ± 1.59	0.75 ± 1.55	0.009*

Values are presented as mean ± standard deviation or number (%).

*$p < 0.05$
